# Effectiveness of Bilateral Arm Training With and Without Rhythmic Auditory Cueing on Functional Mobility of the Upper Limb in Stroke Survivors

**DOI:** 10.7759/cureus.113547

**Published:** 2026-07-28

**Authors:** Yukti K Agrawal, G. Varadharajulu, Suraj Kanase

**Affiliations:** 1 Department of Neurosciences, Krishna College of Physiotherapy, Krishna Vishwa Vidyapeeth (Deemed to be University), Karad, IND

**Keywords:** action research arm test, batrac, bilateral arm training, fugl-meyer assessment, rhythmic auditory cueing, stroke rehabilitation, upper limb

## Abstract

Background: Stroke commonly results in upper limb motor impairment, leading to reduced functional independence and quality of life. Bilateral arm training (BAT) and bilateral arm training with rhythmic auditory cueing (BATRAC) are rehabilitation approaches used to improve upper limb function following stroke. However, the additional benefit of incorporating RAC into BAT requires further investigation.

Objective: To compare the effects of BAT with and without RAC on functional mobility of the upper limb in stroke survivors.

Methods: This experimental study enrolled 24 stroke survivors (age 30-50 years; Brunnstrom stage ≥3; Fugl-Meyer Assessment [FMA] arm score 23-47; stroke duration three or more months) using consecutive sampling. Participants were allocated to Group A (BAT alone, n=12) or Group B (BAT with RAC, n=12). Both groups received 35-minute structured bilateral upper limb training sessions five days per week for four weeks, with Group B performing tasks synchronized to a progressive metronome tempo (60-80 bpm). Outcome measures included the FMA upper extremity score and the Action Research Arm Test (ARAT), assessed at baseline and post-intervention. Paired t-tests were used for within-group comparisons and unpaired t-tests for between-group comparisons. Statistical significance was set at p<0.05.

Results: Both groups demonstrated statistically significant improvements following the intervention. Group A showed a mean FMA improvement of 6.75 (p=0.0426) and a mean ARAT improvement of 8.67 (p=0.0031). Group B demonstrated greater improvements, with a mean FMA gain of 9.67 (p=0.002) and a mean ARAT gain of 14.59 (p<0.0001). Between-group analysis showed significantly greater improvements in Group B for both FMA (mean difference 2.917; t=3.211; p=0.0040) and ARAT (mean difference 5.917; t=4.410; p=0.0002).

Conclusion: Both BAT alone and BAT combined with RAC significantly improved upper limb motor function in stroke survivors. However, the addition of RAC resulted in significantly greater improvements in both motor impairment and functional performance. These findings support the incorporation of BATRAC as an accessible rehabilitation strategy for enhancing upper limb recovery after stroke.

## Introduction

Stroke is among the most devastating neurological emergencies encountered in modern clinical practice. Defined by the American Heart Association and American Stroke Association as a focal or global neurological dysfunction of vascular origin, lasting more than 24 hours or resulting in death, arising from ischemia or hemorrhage in the brain, spinal cord, or retina, stroke represents a heterogeneous syndrome encompassing ischemic infarction, intracerebral hemorrhage, and subarachnoid hemorrhage, each with distinct pathophysiology, clinical presentation, and prognosis [1,2]. Ischemic stroke, which accounts for approximately 68-80% of all events, results from occlusion of a cerebral artery by thrombosis or embolism, triggering a cascade of excitotoxicity, neuroinflammation, and secondary neuronal injury that collectively determine the extent and permanence of neurological impairment [2]. Hemorrhagic stroke, encompassing intracerebral hemorrhage and subarachnoid hemorrhage, accounts for the remaining 20-32% and is associated with higher early case fatality than its ischemic counterpart [2].

From a global epidemiological perspective, the burden of stroke is staggering. According to the Global Burden of Disease Study 2019, stroke was the second leading cause of death and the third leading combined cause of death and disability worldwide, responsible for an estimated 12.2 million new cases, 101 million prevalent cases, 143 million disability-adjusted life years lost, and approximately 6.55 million deaths in 2019 alone [3]. The World Stroke Organization Global Stroke Fact Sheet 2022 estimated that one in four adults over the age of 25 will experience a stroke in their lifetime, with over 13.7 million new strokes occurring annually [4]. Projections from the 2025 World Stroke Organization update further indicate that by 2050, more than 200 million individuals could be living with stroke globally if current risk factor trajectories are not reversed [5]. Modifiable vascular risk factors - including hypertension, diabetes mellitus, dyslipidemia, atrial fibrillation, obesity, tobacco use, and physical inactivity - collectively account for the vast majority of attributable stroke burden, underscoring the critical importance of both primary prevention and optimized post-stroke rehabilitation [4,5].

In India, the epidemiological picture is particularly alarming. Kamalakannan et al. conducted a systematic review reporting crude stroke incidence rates ranging from 105 to 152 per 100,000 population per year and prevalence estimates spanning 44.29 to 559 per 100,000, with urban populations bearing a disproportionately higher burden than rural communities [6]. Jones et al., in a subsequent systematic review, confirmed that stroke case fatality in India ranges from 18.4% to 41% at one month, and highlighted that a substantially higher proportion of strokes in India occur in individuals below 40 years of age - a phenomenon linked to the high prevalence of rheumatic heart disease, hypercoagulable states, and poorly controlled hypertension [7]. The combination of a high incidence, younger demographic profile, and limited access to advanced acute and rehabilitative care underscores the urgent need for contextually appropriate, cost-effective, and evidence-based rehabilitation strategies tailored to the Indian stroke population [6,7].

Among the sequelae of stroke, upper limb paresis is one of the most clinically prevalent and functionally devastating. Between 55% and 75% of stroke survivors experience some degree of upper limb paresis immediately following the event, and approximately 30-40% are left with chronic, persistent upper limb dysfunction that substantially compromises their capacity for independence in activities of daily living [8,9]. The multidimensional nature of post-stroke upper limb impairment encompasses deficits across muscle strength, motor control, inter-muscular coordination, dexterity, sensation, proprioception, and spasticity, each of which interacts with the others to further impede functional recovery [8,10]. Pan et al. highlighted that the assessment and rehabilitation of post-stroke upper limb function must address all levels of the ICF framework - body functions, activities, and participation - to capture the full scope of recovery and its impact on real-world independence [10]. From a phenomenological perspective, stroke survivors have described their affected limb as feeling 'foreign', 'useless', or 'dead', with the loss of upper limb function profoundly disrupting occupational identity, social participation, and emotional wellbeing [8].

Current rehabilitative strategies for upper limb recovery after stroke encompass a broad spectrum of approaches, including constraint-induced movement therapy, task-specific and task-oriented training, mirror therapy, virtual reality, robotic-assisted therapy, and neuromuscular electrical stimulation [11]. Despite the breadth of available modalities, Pollock et al., in a comprehensive Cochrane systematic review, concluded that the overall evidence base remains heterogeneous, with most trials limited by small sample sizes, high risk of bias, and inconsistent outcome measurement, rendering definitive conclusions regarding the superiority of any single approach difficult [11]. A particularly underexplored dimension of upper limb rehabilitation is the integration of interventions that simultaneously address bimanual coordination deficits and harness the neurophysiological potential of auditory-motor coupling - a gap that the present study sought to address.

Bilateral arm training (BAT) is a rehabilitation paradigm in which both upper limbs perform simultaneous, symmetrical movements, with the theoretical rationale that the movement of the intact limb activates ipsilateral motor cortex and transcallosal pathways, thereby providing indirect neuromotor facilitation to the paretic limb [12,13]. This approach is particularly appealing for individuals with moderate to severe paresis, who may lack sufficient voluntary motor control to engage meaningfully in conventional unilateral task-specific training [13]. Rhythmic auditory cueing (RAC) - also referred to as rhythmic auditory stimulation - is a neurologic music therapy technique that delivers externally paced metronome or musical stimuli to entrain voluntary movement via the auditory-motor neural network, encompassing the basal ganglia-thalamocortical circuit, supplementary motor area, premotor cortex, and cerebellum [14,15]. Schaefer proposed that auditory rhythm serves as an anticipatory timing cue that pre-activates motor circuits before movement initiation, thereby improving the temporal precision, consistency, and efficiency of voluntary movement - a mechanism with direct relevance to upper limb motor relearning after stroke [14].

The combination of bilateral arm training with rhythmic auditory cueing - designated BATRAC - was first systematically evaluated by Whitall et al. in a landmark 2000 study demonstrating significant improvements in paretic arm motor function following six weeks of repetitive BATRAC in chronic hemiparetic stroke survivors [16]. Luft et al. subsequently provided functional MRI evidence that BATRAC promotes significantly greater activation of the contralesional primary motor cortex than dose-matched conventional therapy, indicating that the combined approach facilitates beneficial cortical reorganization through the recruitment of ipsilateral motor pathways [17]. Despite this foundational evidence, comparative trials rigorously evaluating BATRAC versus BAT alone - using standardized, validated outcome measures capturing both impairment and activity levels - remain limited, particularly in the Indian rehabilitation context [6,7]. The present study was designed to address this gap by comparing the effectiveness of four weeks of BAT with and without RAC on functional mobility of the upper limb in stroke survivors, employing the Fugl-Meyer Assessment of the Upper Extremity (FMA-UE) and the Action Research Arm Test (ARAT) as primary outcome measures [18-20].

## Materials and methods

Study design and setting

This was a randomized controlled trial, two-group comparative study conducted at the Department of Neurosciences Physiotherapy, Krishna College of Physiotherapy, Krishna Vishwa Vidyapeeth, Karad, Maharashtra, India. The study was conducted over a period of one year. Ethical approval for the study was obtained from the Institutional Ethics Committee of Krishna Vishwa Vidyapeeth, Karad, Maharashtra, India (Reference No. 265/2025-2026). The study was also registered with the Clinical Trials Registry-India in June 2026 (CTRI No. [CTRI/2026/06/112493]). Trial registration was completed after initiation of participant enrolment; this has been stated here for transparency. Written informed consent was obtained from all participants prior to enrolment.

Sample size calculation

The sample size for the present study was estimated using the formula for comparison of two independent group means:

n = 2 × (Zα/2 + Zβ)² × σ² / d²

where n represents the sample size required in each group, Zα/2 is the standard normal deviate corresponding to the chosen level of significance (1.96 at 5% significance level), Zβ is the standard normal deviate corresponding to the desired study power (0.84 for 80% power), σ is the pooled standard deviation, and d is the expected mean difference between the groups. Based on these considerations and the feasibility of participant recruitment within the study period, a total of 24 participants were enrolled and equally allocated to the two study groups. Consecutive sampling was employed to recruit eligible participants from the outpatient and inpatient physiotherapy departments

Participants and eligibility criteria

A total of 30 potential participants were screened, of whom 24 fulfilled all eligibility criteria and were enrolled in the study. Participants were allocated to Group A (BAT alone, n=12) or Group B (BATRAC, n=12) in consecutive order.

Inclusion Criteria

Male or female stroke survivors aged 30-50 years, diagnosis of ischemic/hemorragic stroke involving the middle cerebral artery territory, affecting either the dominant or non-dominant hemisphere, stroke duration of at least three months (subacute to chronic stage), Brunnstrom stage ≥3 in the affected upper limb, FMA-UE score between 23 and 47 (indicating mild to moderate upper limb motor impairment) [18,20], ability to sit unsupported for at least 30 minutes, presence of some voluntary movement in the affected upper limb.

Exclusion Criteria

Severe cognitive impairment preventing participation in structured rehabilitation, significant visual or auditory impairment interfering with the intervention, severe spasticity in the affected upper limb (Modified Ashworth Scale >2), clinical evidence of apraxia, any concurrent musculoskeletal condition affecting upper limb function (e.g., adhesive capsulitis, fracture, severe shoulder pain), any additional neurological disorder that could influence motor performance or rehabilitation outcomes. Participants receiving any concurrent rehabilitation or therapeutic interventions that could influence upper limb function or study outcomes during the intervention period were excluded. These included Occupational Therapy, acupuncture, kinesiotaping, Ayurvedic treatment, Varma therapy, chiropractic or manual therapies.

Demographic information including age, gender, dominant hand, affected side, type and duration of stroke, and relevant comorbidities were recorded for each participant at baseline using a standardized data collection form.

Participants and randomization

Eligible participants were recruited using consecutive sampling and, after baseline assessment, were randomly allocated in a 1:1 ratio to Group A (BAT) or Group B (BATRAC). Randomization was performed using a computer-generated random sequence prepared by an independent researcher not involved in recruitment, treatment, or assessment. Group allocation was concealed using sequentially numbered, opaque, sealed envelopes, which were opened only after participant enrollment and baseline assessment. Outcome assessments were performed by a trained physiotherapist who was not involved in treatment delivery and was blinded to group allocation. Due to the nature of the interventions, participant and therapist blinding was not feasible.

Outcome measures

Two validated, standardized outcome measures were used to assess upper limb motor function at baseline (pre-intervention) and at the end of the four-week intervention period (post-intervention). All assessments were administered by a trained physiotherapist-assessor who was not involved in delivering the intervention.

FMA-UE

The FMA-UE is the most widely used and psychometrically robust standardized instrument for assessing post-stroke upper extremity motor impairment [20]. Originally developed by Fugl-Meyer et al. in 1975, it evaluates motor control, reflex activity, and coordination through 33 items organized hierarchically in accordance with the Brunnstrom stages of motor recovery, each scored on a 3-point ordinal scale (0 = cannot perform; 1 = partially performs; 2 = performs fully), yielding a maximum motor score of 66 [20]. Higher scores reflect greater motor function. A standardized administration procedure published by Sullivan et al. was followed to ensure procedural consistency and inter-rater reliability [18].

ARAT

The ARAT is a performance-based measure of upper extremity functional ability that evaluates the capacity to perform tasks representative of activities of daily living [19]. It comprises 19 items across four subscales - grasp, grip, pinch, and gross movement - each scored on a 4-point ordinal scale, yielding a maximum total score of 57. Higher scores indicate superior functional task performance. Hsieh et al. established the excellent psychometric properties of the ARAT in stroke patients, reporting an intraclass correlation coefficient >0.98 for inter-rater reliability and strong concurrent validity with the FMA [19]. The combined use of the FMA and ARAT in the present study provided a comprehensive, dual-level assessment of upper limb recovery - the FMA capturing neuromotor impairment at the body function level, and the ARAT reflecting functional task performance at the activity level of the ICF framework.

Intervention protocol

Both groups received bilateral upper limb training sessions of 35 minutes duration (excluding the five-minute warm-up), five days per week for four weeks. The intervention protocol was structured in a progressive manner across the four-week period, with tasks increasing in complexity, object weight, number of repetitions, and functional demand from week to week. The same task sequence was applied to both groups; the only difference was that Group B performed all tasks synchronized to a metronome-delivered auditory beat, while Group A trained in silence.

The warm-up phase in both groups consisted of five minutes of passive range of motion (ROM) and active-assisted ROM exercises for the shoulder, elbow, forearm, wrist, and hand joints. In Group B, the warm-up was also performed in synchrony with the metronome at the week-appropriate tempo. The progressive intervention protocol across all four weeks is summarized in Table 1.

**Table 1:** Progressive Four-Week Intervention Protocol for Both Groups (Group A: BAT; Group B: BATRAC) BAT = Bilateral Arm Training; BATRAC = Bilateral Arm Training with Rhythmic Auditory Cueing; ROM = Range of Motion; ADL = Activities of Daily Life

Week	Warm-up (5 min)	Training Tasks (30-35 min)	Progression Focus
1	Passive ROM + Active-Assisted ROM (BAT: no cue; BATRAC: ~60 bpm)	Bilateral reaching to cones; synchronized towel folding; lifting light objects (cups, soft dumbbells); stacking blocks or cups with both hands	Familiarization; symmetry; controlled simple bilateral movement; timing entrainment (BATRAC)
2	Gentle stretching + Active ROM (BATRAC: 65-70 bpm)	Reaching with increased repetitions; folding larger towels; lifting 0.5-1 kg objects; stacking at varied heights	Increased repetitions; slight resistance; task variety; improved temporal coordination (BATRAC)
3	Shoulder mobility + Active ROM (BATRAC: 70-75 bpm)	Pouring water jug to glass bilaterally; bilateral ball passing on table; simulated ADLs: opening box, buttoning large shirts	Functional integration; multi-step bilateral tasks; rhythmic sequencing to beat (BATRAC)
4	ROM + coordination drills (BATRAC: 75-80 bpm)	Assembling puzzle pieces bilaterally; bilateral utensil tasks (knife + fork simulation); resistance band bilateral pulling (light-medium)	Advanced coordination; resistance integration; fine motor + functional bilateral tasks; rhythmic ADL integration (BATRAC)

For participants in Group B, RAC was delivered using an iPhone 16 smartphone. A YouTube-based rhythmic cueing/metronome video was used to provide a regular external auditory beat during the bilateral arm training sessions. The auditory cue was played through the device speaker, and participants were instructed to perform the bilateral upper limb movements in synchrony with the rhythm. The same mode of cue delivery was used consistently across all intervention sessions to maintain uniformity of training. In Group B (BATRAC), the metronome tempo was set at approximately 60 beats per minute (bpm) in Week 1 to allow familiarization with externally cued rhythmic movement. The tempo was progressively increased to 65-70 bpm in Week 2, 70-75 bpm in Week 3, and 75-80 bpm in Week 4, in keeping with the principle of progressive overload applied to temporal demand. Participants were instructed to initiate and complete each bilateral movement in synchrony with the auditory beat. A trained physiotherapist supervised all sessions to ensure protocol adherence, monitor participant safety, and provide corrective feedback regarding bilateral symmetry and movement quality.

Participant adherence to the intervention was monitored using session attendance records. No adverse events were reported during the intervention period in either group. Participants were asked to refrain from receiving any additional formal physiotherapy for the upper limb during the study period.

Statistical analysis

All data were recorded on standardized data collection sheets, entered into an Excel spreadsheet (Microsoft, Redmond, WA, USA), tabulated, and subjected to statistical analysis. Statistical analysis was performed using GraphPad InStat software, version 3.06 (GraphPad Software Inc., San Diego, CA, USA). Data were entered into Microsoft Excel for tabulation prior to analysis. Descriptive statistics - including mean and standard deviation (SD) - were computed for all continuous variables. A paired t-test was used to evaluate the statistical significance of within-group changes in FMA-UE and ARAT scores from pre- to post-intervention for each group independently. An unpaired t-test was used to compare the magnitude of mean change (post-intervention minus baseline) between the two groups for each outcome measure. A p-value of less than 0.05 was considered statistically significant for all comparisons. All reported p-values are two-tailed. In addition to p-values, effect sizes (Cohen’s d) were calculated to estimate the magnitude of within-group and between-group differences. Effect sizes were interpreted as small (0.2), medium (0.5), and large (0.8).

## Results

A total of 24 participants were enrolled and completed the four-week intervention. Group A (BAT alone) comprised 12 participants, while Group B (BATRAC) comprised 12 participants. The baseline demographic and clinical characteristics of the participants are summarized in Table 2. The majority of participants in both groups were aged 46-50 years (50.0% in Group A and 41.7% in Group B). Most participants were male, accounting for 75.0% of Group A and 83.3% of Group B. Hemorrhagic stroke was more common than ischemic stroke in both groups, occurring in 66.7% of participants in Group A and 75.0% in Group B. Regarding stroke duration, 58.3% of participants in Group A and 50.0% in Group B had experienced stroke for more than three months, while the remaining participants had a stroke duration of more than four months.

**Table 2:** Baseline Demographic and Clinical Characteristics of Participants Data are presented as mean ± standard deviation (SD) for continuous variables and n (%) for categorical variables. Independent t-test was used for comparison of continuous variables between groups, and the Chi-square test/Fisher’s exact test was used for categorical variables, as appropriate. A p-value of <0.05 was considered statistically significant. FMA = Fugl-Meyer Assessment (Upper Extremity); ARAT = Action Research Arm Test.

Variable	Category	Group A (n = 12)	Group B (n = 12)
Age Distribution (years)	30-35	2 (16.7%)	1 (8.3%)
	36-40	2 (16.7%)	4 (33.3%)
	41-45	2 (16.7%)	2 (16.7%)
	46-50	6 (50.0%)	5 (41.7%)
Gender	Male	9 (75.0%)	10 (83.3%)
	Female	3 (25.0%)	2 (16.7%)
Type of Stroke	Hemorrhagic	8 (66.7%)	9 (75.0%)
	Ischemic	4 (33.3%)	3 (25.0%)
Duration Since Stroke	>3 months	7 (58.3%)	6 (50.0%)
	>4 months	5 (41.7%)	6 (50.0%)
Baseline FMA Score	Mean ± SD	31.66 ± 7.88	32.58 ± 7.29
Baseline ARAT Score	Mean ± SD	21.91 ± 6.02	25.66 ± 7.30

Baseline upper limb motor impairment and functional performance were comparable between the two groups. The mean baseline FMA-UE score was 31.66 ± 7.88 in Group A and 32.58 ± 7.29 in Group B, while the mean baseline ARAT score was 21.91 ± 6.02 and 25.66 ± 7.30, respectively. Statistical analysis showed no significant between-group differences in baseline demographic or clinical variables (p>0.05), indicating that the groups were comparable before the intervention (Table 2).

Within-group analysis

Paired t-test analysis revealed statistically significant improvements in both FMA-UE and ARAT scores from baseline to post-intervention within both groups (Table 3). These within-group improvements indicate that both BAT alone and BATRAC are effective interventions for improving upper limb motor function over four weeks.

**Table 3:** Within-group comparison of pre- and post-intervention scores for upper limb functional outcomes in Groups A and B. Data are presented as Mean ± Standard Deviation (SD). Within-group pre- and post-intervention comparisons were analyzed using the paired t-test. A p-value of <0.05 was considered statistically significant. Cohen’s d was calculated to estimate the magnitude of treatment effect, with values of 0.2, 0.5, and 0.8 representing small, medium, and large effects, respectively. FMA-UE = Fugl-Meyer Assessment (Upper Extremity); ARAT = Action Research Arm Test; BAT = Bilateral Arm Training; BATRAC = Bilateral Arm Training with Rhythmic Auditory Cueing.

Outcome Measure	Group	Pre-intervention Mean ± SD	Post-intervention Mean ± SD	Mean Difference	t-value	p-value	Effect Size (Cohen’s *d*)	Interpretation
FMA-UE	Group A (BAT)	31.66 ± 7.88	38.41 ± 7.46	6.75	2.153	0.0426	0.88	Large
	Group B (BATRAC)	32.58 ± 7.29	42.25 ± 6.16	9.67	3.507	0.0020	1.43	Large
ARAT	Group A (BAT)	21.91 ± 6.02	30.58 ± 6.73	8.67	3.323	0.0031	1.36	Large
	Group B (BATRAC)	25.66 ± 7.30	40.25 ± 4.43	14.59	5.913	<0.0001	2.42	Very large

In Group A (BAT alone), the mean FMA-UE score improved from 31.66±7.88 at baseline to 38.41±7.46 at post-intervention, representing a mean improvement of 6.75 points (t=2.153; p=0.0426). The mean ARAT score improved from 21.91±6.02 to 30.58±6.73, a mean improvement of 8.67 points (t=3.323; p=0.0031). Both improvements were statistically significant at the p<0.05 level (Table 3).

In Group B (BATRAC), the improvements were larger in magnitude. The mean FMA-UE score improved from 32.58±7.29 to 42.25±6.16, representing a mean gain of 9.67 points (t=3.507; p=0.002). The mean ARAT score improved from 25.66±7.30 to 40.25±4.43, a mean gain of 14.59 points (t=5.913; p<0.0001). Both improvements were statistically significant, with the ARAT improvement in Group B achieving a high level of statistical significance (p<0.0001). The reduction in standard deviation observed in the post-intervention ARAT scores of Group B (from 7.30 to 4.43) also suggests greater homogeneity of response following the BATRAC intervention (Table 3).

Between-group analysis

Unpaired t-test comparison of the mean change scores between the two groups confirmed the statistical superiority of Group B over Group A for both primary outcome measures (Table 4). These findings demonstrate that the addition of RAC to BAT produced significantly greater improvements in both upper limb motor impairment and functional task performance than BAT alone.

**Table 4:** Between-group comparison of mean change scores in upper limb functional outcomes following the intervention. Data are presented as Mean Change ± Standard Deviation (SD). Between-group comparisons of change scores were analyzed using the independent samples t-test. A p-value of <0.05 was considered statistically significant. Cohen’s d represents the magnitude of difference between Group A and Group B. FMA-UE = Fugl-Meyer Assessment (Upper Extremity); ARAT = Action Research Arm Test; BAT = Bilateral Arm Training; BATRAC = Bilateral Arm Training with Rhythmic Auditory Cueing; N = Number of participants.

Outcome Measure	Group	n	Mean Change ± SD	Mean Difference Between Groups	t-value	p-value	Effect Size (Cohen’s *d*)	Interpretation
FMA-UE	Group A (BAT)	12	6.75 ± 1.76	2.917	3.211	0.0040	1.31	Large
	Group B (BATRAC)	12	9.67 ± 2.60					
ARAT	Group A (BAT)	12	8.67 ± 1.92	5.917	4.410	0.0002	1.81	Very large
	Group B (BATRAC)	12	14.59 ± 4.23					

For the FMA-UE, Group B demonstrated a significantly greater mean improvement than Group A (9.67±2.60 vs. 6.75±1.76; mean difference 2.917; t=3.211; p=0.0040). For the ARAT, Group B again showed a significantly superior mean improvement compared with Group A (14.59±4.23 vs. 8.67±1.92; mean difference 5.917; t=4.410; p=0.0002) (Table 4). The between-group p-values for both outcome measures were well below the pre-specified significance threshold of 0.05, providing robust statistical evidence of the incremental benefit conferred by the addition of RAC to the bilateral training paradigm.

## Discussion

The present study examined whether the augmentation of BAT with RAC produces superior improvements in upper limb motor function over BAT alone in stroke survivors following a four-week intervention. Both groups demonstrated statistically significant within-group improvements in FMA-UE and ARAT scores; however, Group B (BATRAC) exhibited significantly greater gains than Group A (BAT alone) on between-group comparison for both outcome measures. These findings are consistent with the theoretical framework underpinning the BATRAC paradigm and align broadly with the existing evidence base examining BAT and auditory-motor entrainment as complementary mechanisms of post-stroke upper limb motor recovery.

The statistically significant within-group improvements observed in Group A provide evidence supporting the standalone efficacy of BAT as a rehabilitation modality for stroke survivors with mild to moderate upper limb motor impairment. The neurophysiological rationale for these improvements is rooted in the interlimb coupling phenomenon, whereby simultaneous, symmetrical bilateral movements engage the undamaged contralesional hemisphere in the reorganization of neural circuits disrupted by the stroke lesion [12,13]. Mudie and Matyas were among the earliest investigators to demonstrate that the densely hemiplegic upper extremity responds more favourably to bilateral training than to unilateral training, attributing this superiority to the coupling of motor drive from the intact hemisphere to the paretic limb via transcallosal and ipsilateral descending pathways [12]. McCombe Waller and Whitall further elaborated the neurophysiological rationale for BAT, noting that it may be particularly beneficial for individuals with moderate to severe paresis who lack the residual voluntary control required to engage meaningfully in conventional unilateral task-specific training [13]. The progressive task complexity employed in the present study's BAT protocol - spanning simple bilateral reaching in Week 1 to resistance band exercises and simulated activities of daily life (ADL) tasks in Week 4 - would have provided increasing demands on the bilateral motor system across the intervention period, consistent with principles of progressive overload and motor learning [11].

The greater improvement observed in Group B is attributable to the additive neurophysiological contribution of auditory-motor entrainment superimposed upon the bilateral training paradigm. RAC operates through the well-characterised anatomical and functional connectivity between the auditory and motor systems, encompassing the basal ganglia-thalamocortical circuit, supplementary motor area, premotor cortex, and cerebellum [14,15]. These interconnected structures collectively form an auditory-motor network capable of translating rhythmic auditory input into temporally organised, precisely timed motor output. Schaefer provided a mechanistic account of how auditory rhythm functions as an anticipatory temporal cue that pre-activates motor planning circuits before movement onset, thereby improving the temporal precision, consistency, and efficiency of voluntary movement - a property with direct relevance to the quality of motor practice during rehabilitation [14]. In the context of BAT, the external rhythmic framework delivered to Group B participants likely provided a more consistent temporal scaffold for organising and pacing bilateral limb movements, resulting in more precisely timed, efficiently executed, and ultimately more therapeutically effective practice repetitions across the four-week intervention [14,15].

The greater improvement observed in the BATRAC group may also be interpreted in light of previously reported neuroplastic effects of bilateral rhythmic upper limb training after stroke. These findings support the hypothesis that rhythmic bilateral training may facilitate motor recovery by promoting cortical reorganisation, enhancing interhemispheric coupling, and strengthening sensorimotor integration. Similarly, later work by Whitall et al. suggested that bilateral and unilateral arm training may improve upper limb function through differing neuroplastic mechanisms, with rhythmic bilateral training potentially engaging distributed motor networks involved in movement timing, coordination, and motor relearning [16]. Earlier work by Luft et al. demonstrated that repetitive BAT with RAC was associated not only with improvement in upper limb motor performance but also with changes in cortical activation patterns on functional magnetic resonance imaging (fMRI), including increased activation in sensorimotor cortical areas and cerebellar regions implicated in motor planning and execution [17]. The present findings, showing greater gains in FMA-UE and ARAT scores in the BATRAC group, are broadly consistent with these earlier mechanistic observations and may reflect enhanced use-dependent plasticity induced by synchronised bilateral movement practice with external auditory cueing [16,17].

However, this mechanistic interpretation should be made cautiously. Unlike the studies by Luft et al. and Whitall et al., the present study did not include direct neurophysiological or neuroimaging assessments such as functional MRI, transcranial magnetic stimulation, or electrophysiological measures [16,17]. Therefore, although the observed clinical improvements are compatible with previously proposed central mechanisms of BATRAC, the present study cannot directly confirm whether similar cortical reorganisation or changes in motor network activation occurred in our participants.

The findings of the present study are directly supported by a number of prior investigations examining the BATRAC paradigm. Whitall et al. conducted the landmark evaluation of repetitive BAT with RAC in individuals with chronic hemiparetic stroke, demonstrating significant improvements in paretic arm motor function, strength, and range of motion following six weeks of training - improvements that were maintained at eight weeks post-intervention [16]. The FMA and ARAT gains observed in Group B of the present study following a four-week protocol mirror these foundational findings and provide additional evidence supporting the efficacy of BATRAC across different durations of intervention. Luft et al., in a randomised controlled trial published in JAMA, employed functional magnetic resonance imaging to demonstrate that participants who received BATRAC showed significantly greater activation of the contralesional primary motor cortex than those receiving dose-matched conventional therapy, providing direct neuroimaging evidence that the combined intervention promotes beneficial cortical reorganisation through the recruitment of ipsilateral motor pathways [17]. Although the present study did not include neurophysiological outcome measures, the magnitude of FMA improvement in Group B - which is hierarchically structured in accordance with Brunnstrom stages of neuromotor recovery - plausibly reflects similar processes of cortical reorganisation underpinning the observed clinical gains. Y Cha et al. investigated the effects of BAT with RAC specifically over a four-week period - a protocol duration identical to that employed in the present study - and reported that the group receiving cued bilateral training demonstrated greater improvements in upper limb function and movement smoothness compared with the group receiving bilateral training alone, a pattern of results closely paralleling the between-group difference identified here [21]. Shahine and Shafshak evaluated the effects of repetitive BATRAC on both clinical motor outcomes and neurophysiological indices of central motor excitability in chronic stroke patients, reporting significant improvements in motor evoked potential amplitude and latency alongside FMA gains, thereby confirming that the functional benefits of BATRAC are accompanied by measurable electrophysiological correlates of cortical plasticity [22]. Mohamed et al. demonstrated that the integration of RAC into bilateral upper extremity training produced significant improvements in both upper extremity motor function and functional independence in stroke patients, supporting the conclusion that the therapeutic value of BATRAC extends across diverse patient populations and clinical contexts [23].

The ARAT improvements observed in both groups reflect the task-oriented, functionally representative nature of the bilateral exercises employed in the present study protocol. The activities selected - including bilateral reaching to cones, towel folding, object lifting and manipulation, peg board tasks, simulated pouring and buttoning, and resistance band exercises - engage multiple levels of the upper limb motor system simultaneously (shoulder, elbow, wrist, and hand) and closely resemble components of real-world daily living activities. Task-specific and task-oriented training is well established as a primary driver of motor learning and experience-dependent neuroplastic adaptation following stroke [11]. The significantly greater ARAT gains in Group B suggest that RAC additionally enhanced the quality, coordination, and temporal consistency of movement execution during these multi-joint, multi-segment functional tasks, consistent with Whitall and Waller's conclusion that auditory cueing improves the timing, coordination, and amplitude of arm movements by providing an external temporal reference that compensates for impaired internal timing mechanisms in the stroke-affected motor system [24].

The transdiagnostic evidence base for RAC lends further mechanistic support to the present findings. Yoo and Kim's systematic review and meta-analysis demonstrated that RAC significantly improves motor performance, coordination, and movement timing in stroke populations, establishing a robust empirical foundation for its incorporation into upper limb rehabilitation protocols [15]. Wei Fan et al. extended this evidence to Parkinson's disease, reporting significant improvements in upper limb movement speed, amplitude, and rhythmicity following rhythmic auditory stimulation, further validating the capacity of auditory-motor coupling to enhance motor output independent of the specific underlying neurological diagnosis [25]. Ghai et al., in a systematic review and meta-analysis of the effects of RAC on gait in ageing populations, demonstrated robust and consistent improvements in spatiotemporal gait parameters, reinforcing the broad neurophysiological validity of rhythm-driven motor entrainment as a rehabilitation strategy across different patient populations and movement domains [26]. A recent randomised, double-blind controlled trial directly comparing bilateral and unilateral upper limb training augmented with RAC in chronic stroke patients found significantly greater FMA-UE improvements and more favourable changes in interhemispheric inhibition in the bilateral group, offering contemporary and methodologically rigorous neurophysiological corroboration for the bilateral training principle adopted in both arms of the present study [27]. The reduction in excessive transcallosal inhibition from the contralesional to the ipsilesional hemisphere proposed as a mediating mechanism provides a plausible physiological explanation for the superior functional outcomes associated with rhythmically cued bilateral training observed in the present study [27].

The motivational and attentional dimensions of RAC also merit consideration as contributing factors to the between-group difference. The presence of an external rhythmic stimulus may have served not only as a precise motor timing cue but also as an engaging and motivating element of the therapy session, potentially enhancing participant attention, effort allocation, and adherence to the prescribed bilateral movement pattern across the four-week intervention. Altenmuller and James, reviewing the impact of music-based interventions in post-stroke motor rehabilitation, observed that musical rhythm engages distributed auditory and motor neural circuits simultaneously, providing a richly integrated sensorimotor experience that conventional physical training cannot replicate, and noted that this multisensory engagement may itself contribute to improved adherence and therapeutic outcomes [28]. While the present study did not formally quantify participant motivation or engagement, the consistently smaller post-intervention standard deviations in Group B - particularly for the ARAT - may indirectly reflect greater training consistency and engagement in participants who received the auditory cueing stimulus.

The broader significance of the present findings is further reinforced by recent systematic evidence on rehabilitation strategies for upper limb impairment after stroke. Doumen et al., evaluating rehabilitation strategies for severe upper limb impairment in early stroke, concluded that intensive and targeted interventions including bilateral training demonstrate benefits for motor recovery, while acknowledging that the overall quality of evidence remains low to moderate and calling for larger, well-designed comparative trials to strengthen the evidence base [29]. Richards et al. similarly reported that BAT produced greater gains in upper limb function and movement symmetry than unilateral arm training in patients with severe arm paresis, reinforcing the rationale for bilateral training as the common rehabilitation platform for both groups in the present study [30]. These convergent findings from the recent literature collectively validate the study design and underscore the clinical relevance of the present results.

The contextual applicability of these findings to the Indian stroke rehabilitation landscape deserves particular emphasis. As documented by Kamalakannan et al. and Jones et al., stroke survivors in India are younger on average than those typically represented in Western rehabilitation trials, present with more heterogeneous stroke aetiology, and face significant socioeconomic and logistical barriers to accessing specialised rehabilitation services and advanced rehabilitation technologies [6,7]. The materials used in the present study - cones, towels, lightweight household objects, resistance bands, a peg board, and a freely downloadable metronome application - are inexpensive, widely available across both urban and rural clinical settings, and require minimal specialised infrastructure or technical expertise to administer. The demonstration that the addition of such a low-cost, easily implementable rhythmic auditory cue to BAT produces a statistically significant and clinically meaningful additional benefit over bilateral training alone has direct and immediate implications for the design of accessible, scalable, and evidence-based upper limb rehabilitation protocols suited to the full spectrum of clinical settings encountered across India [6,7,11].

The findings of the present study must also be interpreted in the context of the important observations made by Richardson et al., who reported that BATRAC was not uniformly efficacious across all stroke survivors and emphasised the need to identify patient-specific factors - including stroke chronicity, lesion characteristics, and baseline motor function - that predict individual responsiveness to the combined intervention [30]. The relatively homogeneous eligibility criteria adopted in the present study - specifically, participants with Brunnstrom stage 3 or above, FMA arm scores between 23 and 47, and stroke duration of at least three months - may have contributed to the consistent positive response observed in Group B by selecting a population of moderately impaired, subacute-to-chronic stroke survivors who retained sufficient voluntary motor control to benefit from rhythmically paced bilateral practice, while appropriately excluding individuals with severe spasticity, apraxia, or cognitive impairment who might respond less favourably [30]. Specifically, Richards et al. tested a modified condensed form of BATRAC in chronic stroke participants and found that, while the intervention facilitated increased use of the paretic arm, improvements in objective motor performance were not consistently replicated across the sample, suggesting that the therapeutic response to BATRAC may vary depending on individual patient characteristics such as baseline motor severity, lesion topography, and residual corticospinal tract integrity [31].

Several limitations of the present study should be acknowledged while interpreting the findings. First, the sample size was relatively small (n = 24), which may limit the statistical power of the study and reduce the generalisability of the findings to the broader stroke population. Although statistically significant differences and large effect sizes were observed, the small sample increases the possibility that the estimated treatment effects may be unstable and should therefore be interpreted with caution. Second, the study was conducted at a single tertiary-care physiotherapy centre, which may limit the external validity of the results, as patient characteristics, rehabilitation practices, and resource availability may differ across institutions and regions. Third, the intervention period and outcome assessment were limited to four weeks, and no long-term follow-up was performed. As a result, it is not possible to determine whether the observed improvements in upper limb motor function and task performance were sustained beyond the immediate post-intervention period. The absence of long-term outcomes also limits conclusions regarding retention of motor gains, transfer to activities of daily living, and the durability of the added benefit of RAC over time. In addition, neurophysiological outcome measures such as motor evoked potentials or functional neuroimaging were not included, precluding direct assessment of the cortical and subcortical mechanisms underlying the observed clinical improvements. Future studies should therefore employ larger, multicentre randomised controlled designs with longer follow-up durations and inclusion of long-term functional and neurophysiological outcomes to confirm the reproducibility, sustainability, and clinical applicability of BATRAC in stroke rehabilitation.

## Conclusions

Both BAT alone and in combination with RAC significantly improve upper limb motor function and functional task performance in stroke survivors over four weeks. The addition of RAC to BAT confers significantly greater improvements in both FMA and ARAT scores, supporting the superiority of the BATRAC approach. Given the low cost and ready availability of the materials required, BATRAC represents a clinically practical, evidence-based enhancement to bilateral upper limb rehabilitation that is particularly well-suited to resource-constrained settings such as those prevalent across India. Future larger, randomized, controlled, multi-center trials with longer follow-up and neurophysiological outcome measures are warranted to further characterize the optimal dosing parameters, patient subgroups, and durability of benefit associated with this combined approach.
